# Association between maternal country of birth and preterm birth: A population-based register study of 910,752 deliveries

**DOI:** 10.1177/1403494821992894

**Published:** 2021-02-15

**Authors:** Tiril Tingleff, Sari Räisänen, Åse Vikanes, Leiv Sandvik, Katariina Laine

**Affiliations:** 1 Institute of Clinical Medicine, Faculty of Medicine, University of Oslo, Norway; 2 Tampere University of Applied Sciences, Finland; 3 Gynklinikk Nydalen AS, Norway; 4 Department of Obstetrics, Oslo University Hospital, Norway

**Keywords:** Epidemiology, extremely preterm birth, immigrant, late preterm birth, medical birth registry, preterm birth, socio-economic factors, very preterm birth

## Abstract

**Aims:** The aim of this study was to analyse associations between maternal country of birth and preterm birth among women giving birth in Norway. **Methods:** A population-based register study was conducted employing official national databases in Norway. All singleton births, with neonates without major anomalies, between 1999 and 2014 were included (*N*=910,752). We estimated odds ratios (ORs) for extremely preterm birth (<28 weeks gestation), very preterm birth (28–33 weeks gestation) and late preterm birth (34–36 weeks gestation) by maternal country of birth. We conducted multivariable regression analyses, adjusting for maternal, obstetric and socio-economic confounders. **Results:** For extremely preterm births (0.4% of the study population), women with an unknown country of birth (adjusted OR (aOR)=3.09; 95% confidence interval (CI) 2.26–4.22) and women born in sub-Saharan Africa (aOR=1.66; CI 1.40–1.96) had the highest ORs compared to Norwegian-born women. For very preterm births (1.2% of the study population), women with an unknown country of birth (aOR=1.72; CI 1.36–2.18) and women born in South Asia (aOR=1.48; CI 1.31–1.66) had the highest ORs. For late preterm births (3.8% of the study population), women born in East Asia Pacific/Oceania (aOR=1.33; CI 1.25–1.41) and South Asia (aOR=1.30; CI 1.21–1.39) had the highest ORs. **Conclusions: After adjusting for maternal, obstetric and socio-economic risk factors, maternal country of birth remained significantly associated with preterm birth. Women with an unknown country of birth and women born in sub-Saharan Africa were found to be at increased risk of extremely preterm birth.**

## Introduction

Preterm birth, defined as birth before 37 weeks’ gestation, is a major cause of neonatal mortality and morbidity worldwide. National preterm birth rates vary in the range of 5–18%, with low-income countries having the highest rates [1]. Preterm birth is considered a multifactorial syndrome related to numerous maternal, obstetric and psychosocial risk factors [2,3]. Gestational age at delivery and quality of care in the neonatal period affect infant outcomes, with the highest risks for severe complications being observed for newborns delivered at the lowest gestational ages [4,5].

Immigration, which is increasing worldwide, may be considered to be a composite social health determinant, with individual, cultural and structural factors affecting health outcomes [6]. Whereas 16% of women giving birth in Norway were foreign born in 1999, 29% were foreign born in 2014. This increase includes immigrants from all continents. Immigrant women from various world regions have been reported to be at increased risk of preterm birth [7,8]. However, to the best of our knowledge, large population-based studies of associations between maternal country of birth and extremely preterm birth are lacking.

The main aim of the study was to assess associations between extremely, very and late preterm birth and maternal country of birth among women giving birth in Norway. A secondary aim was to explore the effects of maternal, obstetric and socio-economic factors on associations between preterm birth and maternal country of birth.

## Methods

This was a population-based register study. Data obtained from two national registries – the Medical Birth Registry of Norway (MBRN) and Statistics Norway (SSB) – were merged. The MBRN was established in 1967, where registration of data on maternal and neonatal health became mandatory for all deliveries in Norway, including home births. Information is collected consecutively throughout pregnancy and birth. Midwives complete the notification to the MBRN when discharging the women after birth. SSB compiles official statistics about Norwegian society, including the country of birth and education levels of the residents of Norway.

The study sample consisted of 930,684 births in the gestational age range of 22–44 weeks that were registered between 1999 and 2014. Multiple births and births in which neonates had major anomalies were excluded, leaving a total sample size of 910,752 births. Since 1999, gestational age in the MBRN has been based on second-trimester ultrasound examination markers. More than 97% of pregnant women in Norway undergo an ultrasound examination between gestational weeks 17 and 20 offered free of charge. The outcome variable, preterm birth, was divided into three subgroups: extremely preterm, birth before gestational week 28; very preterm, birth between gestational week 28^+0^ and 33^+6^; and late preterm, birth between gestational week 34^+0^ and 36^+6^. The categorisation was based on clinical relevance. In line with World Health Organization, birth before week 28 is termed extremely preterm. Babies born extremely preterm have the highest mortality and morbidity risk. In contrast, babies born after week 33 are considered low risk. Norwegian clinical guidelines recommend antenatal corticosteroids and tocolytics until the end of week 33.

The main independent risk factor was maternal country of birth. Countries were divided into nine regions based on the World Bank’s classification scheme. The reference group consisted of women born in Norway. Europe was divided into countries in the European Economic Association (EEA), including Switzerland, and countries outside the EEA. Our sample included relatively low numbers of women from Transcaucasia, Central Asia and Oceania. Based on demographic factors, women born in Transcaucasia and Central Asia regions were merged into a single group with women born in non-EEA European countries. Women born in countries within the East Asia or Oceania regions were merged into a single East Asia Pacific/Oceania group (Table I).

**Table I.** Prevalence of preterm and term deliveries by maternal characteristics (*N*=910,752).

Maternal characteristic	Extremely preterm	Very preterm	Late preterm	Term
	0.4% (*N*=3429)	1.2% (*N*=10,952)	3.8% (*N*=34,206)	94.7% (*N*=862,165)
Birthplace, by region				
Norway (*N*=727,054)	0.4% (2582)	1.2% (8638)	3.7% (27,163)	94.7% (688,671)
Europe, EEA^a^ (*N*=55,341)	0.4% (194)	1.0% (546)	3.3% (1825)	95.4% (52,776)
Europe, non-EEA/Transcaucasia/Central Asia^b,c^ (*N*=22,152)	0.3% (77)	1.1% (237)	3.4% (748)	95.2% (21,090)
North America^d^ (*N*=4000)	0.4% (14)	1.1% (43)	2.8% (112)	95.8% (3831)
Latin America/Caribbean^e^ (*N*=7867)	0.6% (44)	1.2% (91)	4.2% (330)	94.1% (7402)
Middle East/North Africa^f^ (*N*=19,654)	0.4% (86)	1.3% (249)	3.8% (741)	94.5% (18,578)
Sub-Saharan Africa^g^ (*N*=24,148)	0.7% (176)	1.5% (367)	3.4% (819)	94.4% (22,786)
South Asia^h^ (*N*=19,478)	0.6% (108)	1.7% (324)	4.9% (956)	92.9% (18,090)
East Asia Pacific/Oceania^i,j^ (*N*=26,068)	0.3% (83)	1.4% (360)	4.9% (1290)	93.4% (24,335)
Unknown country of birth (*N*=4990)	1.3% (65)	1.9% (97)	4.4% (222)	92.3% (4606)
Educational level				
None/primary education (*N*=149,234)	0.5% (768)	1.6% (2324)	4.5% (6757)	93.4% (139,385)
Secondary education (*N*=273,222)	0.4% (1081)	1.3% (3567)	4.0% (11,013)	94.3% (257,561)
Bachelor (*N*=344,095)	0.3% (1050)	1.0% (3572)	3.4% (11,667)	95.3% (327,806)
Master’s/PhD (*N*=108,402)	0.3% (316)	0.9% (1009)	3.1% (3364)	95.7% (103,713)
Missing (*N*=35,799)	0.6% (214)	1.3% (480)	3.9% (1405)	94.1% (33,700)
Marital status				
Married/cohabiting/partner (*N*=839,760)	0.4% (2961)	1.2% (9745)	3.7% (30,783)	94.8% (796,271)
Single/widowed (*N*=70,992)	0.7% (468)	1.7% (1207)	4.8% (3423)	92.8% (65,894)
Maternal age (years)				
<20 (*N*=19,880)	0.6% (120)	1.8% (366)	5.3% (1054)	92.3% (18,340)
20–24 (*N*=133,904)	0.4% (520)	1.2% (1648)	4.1% (5466)	94.3% (126,270)
25–29 (*N*=296,069)	0.3% (948)	1.1% (3303)	3.6% (10,723)	94.9% (281,095)
30–34 (*N*=301,144)	0.3% (1026)	1.1% (3354)	3.5% (10,424)	95.1% (286,340)
35–39 (*N*=135,184)	0.5% (656)	1.4% (1845)	3.9% (5328)	94.2% (127,355)
⩾40 (*N*=24,502)	0.6% (156)	1.8% (436)	4.9% (1203)	92.7% (22,707)
First-trimester smoking				
No/missing (*N*=790,495)	0.4% (2881)	1.2% (9094)	3.7% (28,854)	94.8% (749,666)
Sometimes (*N*=14,554)	0.4% (56)	1.4% (197)	3.7% (534)	94.6% (13,767)
Daily (*N*=105,703)	0.5% (492)	1.6% (1661)	4.6% (4818)	93.4% (98,732)
Chronic hypertension (*N*=5107)	1.8% (94)	4.8% (246)	8.8% (450)	84.5% (4317)
Diabetes				
Type 1 (*N*=4311)	0.7% (32)	5.1% (222)	17.7% (761)	76.5% (3296)
Type 2 (*N*=1948)	0.7% (13)	3.0% (58)	10.7% (209)	85.6% (1668)
Gestational diabetes (*N*=14,247)	0.2% (35)	1.7% (245)	7.0% (1002)	91.0% (12,965)
PE/eclampsia/HELLP (*N*=31,808)	1.7% (540)	8.6% (2751)	14.2% (4522)	75.4% (23,995)
Gestational hypertension (*N*=15,923)	0.3% (54)	1.5% (236)	5.3% (840)	92.9% (14,793)
Placenta previa (*N*=2634)	1.4% (38)	12.3% (323)	27.7% (730)	58.6% (1543)
IVF treatment (*N*=18,411)	0.7% (128)	2.2% (407)	5.4% (995)	91.7% (16,881)
Parity				
Nulliparous (*N*=384,270)	0.4% (1699)	1.5% (5642)	4.4% (16,808)	93.7% (360,121)
Para 1 (*N*=329,940)	0.3% (921)	0.9% (3005)	3.1% (10,244)	95.7% (315,770)
Para >1 (*N*=196,542)	0.4% (809)	1.2% (2305)	3.6% (7154)	94.8% (186,274)
Previous stillbirth (*N*=7395)	1.2% (92)	3.5% (258)	8.9% (657)	86.4% (6388)
Previous second-trimester abortion (*N*=23,783)	1.2% (296)	2.1% (502)	5.0% (1188)	91.6% (21,797)

a Europe, EEA: Sweden, Finland, Denmark, Iceland, Cyprus, Bulgaria, Estonia, Croatia, Latvia, Poland, Romania, Lithuania, Slovenia, Hungary, Slovakia, Czech Republic, Belgium, France, Greece, Ireland, Italy, Malta, The Netherlands, Liechtenstein, Luxembourg, Portugal, Spain, the UK, Switzerland (not actually in the EEA), Germany, Austria.

b Europe, non-EEA: Greenland, Faroe Islands, Albania, Belarus, Moldova, Russia, Turkey, Ukraine, Bosnia-Herzegovina, Macedonia, Serbia, Montenegro, Kosovo, Andorra, Gibraltar, Monaco, San Marino, Vatican City State, Guernsey, Jersey, Isle of Man.

c Transcaucasia/Central Asia: Armenia, Azerbaijan, Georgia, Kazakhstan, Kyrgyzstan, Tajikistan, Turkmenistan, Uzbekistan.

d North America: Canada, Saint Pierre and Miquelon, USA.

e Latin American/Caribbean: US Virgin Islands, Barbados, Antigua and Barbuda, Belize, Bahamas, Bermuda, British Virgin Islands, Cayman Islands, Costa Rica, Cuba, Dominica, Dominican Republic, Grenada, Guadeloupe, Guatemala, Haiti, Honduras, Jamaica, Martinique, Mexico, Montserrat, Aruba, Sint Maarten, Bonaire, Sint Eustatius and Saba, Anguilla, Curaçao, Nicaragua, Panama, El Salvador, Saint Kitts og Nevis, Saint Lucia, Saint Vincent and the Grenadines, Trinidad and Tobago, Turks and Caicos Islands, Puerto Rico, Saint Martin, Saint Barthélemy, Argentina, Bolivia, Brazil, Guyana, Chile, Colombia, Ecuador, Falkland Islands, French Guiana, Paraguay, Peru, Suriname, Uruguay, Venezuela.

f Middle East/North Africa: Algeria, Egypt, Djibouti, Libya, Morocco, Tunisia, Bahrain, United Arab Emirates, Iraq, Iran, Israel, Jordan, Kuwait, Lebanon, Oman, Palestine, Qatar, Saudi Arabia, Syria, Yemen.

g Sub-Saharan Africa: Angola, Botswana, Saint Helena, Burundi, Comoros, Benin, Equatorial Guinea, Côte d’Ivore, Eritrea, Ethiopia, Gabon, Gambia, Ghana, Guinea, Guinea-Bissau, Cameroon, Cape Verde, Kenya, Congo-Brazzaville, Congo, Lesotho, Liberia, Madagascar, Malawi, Mali, Western Sahara, Mauritania, Mauritius, Namibia, Niger, Nigeria, Mozambique, Mayotte, Réunion, Zimbabwe, Rwanda, Sao Tome and Principe, Senegal, Central African Republic, Seychelles, Sierra Leone, Somalia, South Sudan, Sudan, Swaziland, South Africa, Tanzania, Chad, Togo, Uganda, Zambia, Burkina Faso.

h South Asia: British Indian Ocean Territory, Afghanistan, Bangladesh, Bhutan, Sri Lanka, India, Maldives, Nepal, Pakistan.

i East Asia Pacific: Brunei, Myanmar, Philippines, Taiwan, Hong Kong, Indonesia, Japan, Cambodia, China, North Korea, South Korea, Laos, Macao, Malaysia, Mongolia, Timor-Leste, Singapore, Thailand, Vietnam, Solomon Islands, Fiji, Vanuatu, Tonga, Kiribati, Tuvalu, Nauru, Federated States of Micronesia, Papua New Guinea, Samoa, Marshall Islands, Palau.

j Oceania: American Samoa, Australia, Christmas Island, Cocos (Keeling) Islands, Cook Islands, French Polynesia, Guam, United States Minor Outlying Islands, New Zealand, Niue, Norfolk Island, Pitcairn, Tokelau, Wallis and Futuna Islands, New Caledonia, Northern Mariana Islands.

PE: pre-eclampsia; IVF: in vitro fertilisation.

Risk factors for preterm birth were accounted for in analysis adjustments. Education and marital status were used as proxies for socio-economic status. The eight levels of education in the 2011 International Standard Classification of Education were simplified into four levels according to number of years of education. Women who completed some secondary education (11–14 years) were a reference group. Marital status was dichotomised into married/cohabiting/partner or single/widowed.

Maternal age at the time of birth was divided into five groups with five-year intervals, with the 25- to 30-year-old age group serving as a reference group. First-trimester smoking was categorised as sometimes, daily and non-smoking. Patients with diabetes were designated as type 1, type 2 or gestational diabetes. Patients were designated as having preeclampsia, eclampsia, or HELLP syndrome or not, forming a single dichotomous variable. Parity was grouped into nulliparous, para 1 and para ⩾2; para 1 was used as the reference group. Gestational hypertension, placenta previa, in vitro fertilisation and obstetric history of a stillbirth or second-trimester abortion were considered conditions with possible associations with preterm birth. These variables were treated as dichotomous categories.

### Missing data

Information on smoking was missing for 16% of the cases. We categorised these cases as non-smokers, in line with previous studies based on MBRN data [9]. Information on education was missing in 3.9% of the study population. To avoid excluding these cases, this group was managed as a separate category in all analyses. Maternal country of birth was missing in 0.5% of the study population. These women were included in a category termed ‘unknown country of birth’. The frequency of missing data was <1% for all other variables.

### Ethical approval and patient consent

This population-based cohort study is part of the PURPLE Study approved by the Regional Committee for Medical Research Ethics in South East Norway in 2015 (2015/681) and the Institutional Personal Data Officer in Oslo University Hospital. The study was conducted in accordance with the Norwegian Health Research legislation. The researchers received only anonymised data.

### Statistical analyses

Continuous data were categorised. Crude logistic regression analyses were performed to identify significant risk factors, which were included in subsequent multivariable regression analyses. Two models encompassing maternal, obstetric and socio-economic variables were evaluated. Model 1 included maternal morbidity, age, obstetric history and pregnancy-related conditions. Model 2 consisted of the model 1 constituents plus socio-economic factors. We performed a separate multivariable regression analysis and obtained adjusted odds ratio (aORs) for each outcome. Births occurring before week 28 were excluded in our analyses of the very preterm birth group, and only births occurring after the start of week 34 were included in our analyses of the late preterm birth group. To assess how well study factors in each model explained outcome differences, we calculated the percentage differences in the models with the formulas (OR–aOR1)/(OR–1) and (aOR1–aOR2)/(aOR1–1). No interactions or multicollinearity were observed between independent variables. IBM SPSS Statistics for Windows v25 (IBM Corp., Armonk, NY) was used to perform the statistical analyses.

## Results

### Study population

Maternal characteristics by gestational age at birth are shown in Table I. About one fifth of the women in our study population (*n*=183,698; 20.2%) were not born in Norway. The overall preterm birth rate for gestational age <37 weeks (i.e. extremely+very+late) was 5.3% (*n*=48,587). Overall, the preterm birth rate was highest among women with an unknown country of birth (7.7%), women born in South Asia (7.1%) and women born in East Asia Pacific/Oceania (6.6%). Among Norwegian-born women, the preterm birth rate was 5.3%.

### Extremely preterm birth

The extremely preterm birth rate (<28 gestational weeks) was 0.4% (*n*=3429) for the study population. Extremely preterm rates were highest among women with an unknown country of birth (1.3%) and women born in sub-Saharan Africa (0.7%; Table I). Compared to Norwegian-born women, we observed the greatest OR increases for extreme preterm birth among women with an unknown country of birth (OR=3.70; 95% confidence interval (CI) 2.89–4.74) and for women born in sub-Saharan Africa (OR=2.06; 95% CI 1.77–2.40; Table II). Adjusting for maternal and obstetric risk factors (model 1) increased the extremely preterm birth OR for women with an unknown country of birth and women born in sub-Saharan Africa by around 8%. Comparing models 1 and 2 revealed that education and marital status explained 28.2% of the increased extremely preterm birth ORs among women with an unknown country of birth and 42.6% of the increased ORs among women born in sub-Saharan Africa.

**Table II.** Associations of factors with extremely preterm birth (*N*_group_=3429) reported as crude and adjusted ORs with 95% CIs (*N*_total_=910,752).

Maternal characteristic	Extremely preterm birth < 28^+0^ weeks					
	Crude analyses		Multivariable regression analyses, model 1		Multivariable regression analyses, model 2	
	OR	95% CI	aOR_1_	95% CI	aOR_2_	95% CI
Birthplace, by region						
Norway	Ref.		Ref.		Ref.	
Europe, EEA	0.99	0.85–1.14	1.02	0.88–1.18	1.01	0.87–1.17
Europe, non-EEA/Transcaucasia/Central Asia	0.98	0.78–1.23	1.04	0.83–1.31	0.97	0.77–1.21
North America	0.99	0.58–1.67	0.97	0.57–1.65	0.99	0.58–1.67
Latin America/Caribbean	1.58	1.17–2.13	1.60	1.18–2.16	1.44	1.06–1.94
Middle East/North Africa	1.23	0.99–1.53	1.35	1.09–1.68	1.18	0.94–1.47
Sub-Saharan Africa	2.06	1.77–2.40	2.15	1.84–2.51	1.66	1.40–1.96
South Asia	1.56	1.29–1.90	1.78	1.47–2.17	1.56	1.28–1.91
East Asia Pacific/Oceania	0.90	0.72–1.12	0.97	0.78–1.21	0.87	0.70–1.09
Unknown country of birth	3.70	2.89–4.74	3.91	3.03–5.05	3.09	2.26–4.22
Educational level						
None/primary education	1.30	1.19–1.43			1.17	1.06–1.30
Secondary education	Ref.				Ref.	
Bachelor	0.77	0.71–0.84			0.79	0.73–0.87
Master’s/PhD	0.74	0.65–0.83			0.74	0.65–0.84
Missing	1.51	1.31–1.75			1.14	0.94–1.34
Marital status						
Married/cohabiting/partner	Ref.				Ref.	
Single/widowed	1.88	1.70–2.07			1.55	1.40–1.72
Maternal age (years)						
<20	1.89	1.56–2.29	1.52	1.26–1.85	1.12	0.92–1.37
20–24	1.21	1.10–1.35	1.10	0.98–1.22	0.98	0.87–1.09
25–29	Ref.		Ref.		Ref.	
30–34	1.06	0.97–1.16	1.12	1.02–1.23	1.17	1.07–1.28
35–39	1.52	1.37–1.68	1.51	1.36–1.67	1.56	1.41–1.74
⩾40	2.00	1.68–2.36	1.80	1.51–2.15	1.82	1.53–2.17
First-trimester smoking						
No/missing	Ref.		Ref.			
Sometimes	1.06	0.81–1.38	1.12	0.86–1.46	1.04	0.79–1.35
Daily	1.28	1.16–1.41	1.37	1.24–1.51	1.17	1.05–1.29
Chronic hypertension	5.07	4.13–6.24	3.15	2.54–3.90	3.10	2.50–3.84
Diabetes						
Type 1	1.98	1.40–2.81	1.30	0.91–1.85	1.27	0.89–1.81
Type 2	1.78	1.03–3.07	1.00	0.58–1.74	0.94	0.54–1.64
Gestational diabetes	0.65	0.47–0.91	0.47	0.33–0.65	0.46	0.33–0.64
PE/eclampsia/HELLP	5.24	4.77–5.75	4.76	4.32–5.23	4.69	4.26–5.16
Gestational hypertension	0.90	0.69–1.18	1.01	0.77–1.33	1.02	0.78–1.33
Placenta previa	3.91	2.83–5.39	3.71	2.68–5.13	3.65	2.64–5.06
IVF treatment	1.89	1.58–2.25	1.62	1.35–1.94	1.65	1.37–1.98
Parity						
Nulliparous	1.59	1.46–1.72	1.52	1.40–1.66	1.55	1.42–1.68
Para 1	Ref.		Ref.		Ref.	
Para >1	1.48	1.34–1.62	1.20	1.08–1.32	1.14	1.04–1.26
Previous stillbirth	3.40	2.76–4.19	2.81	2.26–3.49	2.79	2.25–3.47
Previous second-trimester abortion	3.56	3.15–4.01	3.42	3.03–3.87	3.38	2.99–3.82

Model 1: Adjusted for all the variables except education and marital status.

Model 2: Adjusted for all the variables included education and marital status.

OR: odds ratio; CI: confidence interval.

Women born in South Asia had a 1.6-fold increased extremely preterm birth OR compared to Norwegian-born women, with 28.2% of the increased OR in model 1 being explained by education and marital status in model 2.

### Very preterm birth

The very preterm birth rate (between 28^+0^ and 33^+6^ gestational weeks) was 1.2% (*n*=10,952) for the study population. Very preterm birth rates were highest among women with an unknown country of birth (1.9%) and women born in South Asia (1.7%). Compared to Norwegian-born women, we observed the greatest OR increases for very preterm birth among women with an unknown country of birth (OR=1.67; 95% CI 1.36–2.04) and women born in South Asia (OR=1.41; 95% CI 1.26–1.58; Table III). Adjusting for maternal and obstetric risk factors (model 1) increased the ORs for very preterm birth for women with an unknown country of birth by 40.2% and for women born in South Asia by 56.0%. Comparing models 1 and 2 revealed that education and marital status explained 23.4% of the increased very preterm birth OR among women with an unknown country of birth and 25.0% of the increased OR among women born in South Asia.

**Table III.** Associations of factors with very preterm birth (*N*_group_=10,952) reported as crude and adjusted ORs with 95% CIs (*N*_total_=907,323).

Maternal characteristic	Very preterm birth 28^+0^ to 33^+6^ weeks					
	Crude analyses		Multivariable regression analyses, model 1		Multivariable regression analyses, model 2	
	OR	95% CI	aOR_1_	95% CI	aOR_2_	95% CI
Birthplace, by region						
Norway	Ref.		Ref.		Ref.	
Europe, EEA	0.83	0.76–0.90	0.90	0.82–0.98	0.89	0.82–0.98
Europe, non-EEA/Transcaucasia/Central Asia	0.90	0.79–1.02	1.01	0.89–1.15	0.95	0.84–1.09
North America	0.90	0.67–1.22	0.96	0.71–1.30	0.98	0.72–1.33
Latin America/Caribbean	0.98	0.79–1.20	1.00	0.81–1.24	0.94	0.76–1.16
Middle East/North Africa	1.07	0.94–1.21	1.27	1.11–1.44	1.14	1.00–1.31
Sub-Saharan Africa	1.29	1.16–1.43	1.38	1.24–1.54	1.17	1.04–1.31
South Asia	1.41	1.26–1.58	1.64	1.46–1.83	1.48	1.31–1.66
East Asia Pacific/Oceania	1.16	1.05–1.30	1.27	1.14–1.42	1.18	1.05–1.31
Unknown country of birth	1.67	1.36–2.04	1.94	1.58–2.38	1.72	1.36–2.18
Educational level						
None/primary education	1.20	1.14–1.26			1.14	1.08–1.21
Secondary education	Ref.				Ref.	
Bachelor	0.79	0.76–0.83			0.82	0.78–0.86
Master’s/PhD	0.71	0.66–0.76			0.75	0.70–0.81
Missing	1.03	0.94–1.13			1.03	0.92–1.16
Marital status						
Married/cohabiting/partner	Ref.				Ref.	
Single/widowed	1.48	1.39–1.57			1.24	1.17–1.33
Maternal age (years)						
<20	1.67	1.50–1.86	1.31	1.17–1.47	1.06	0.95–1.20
20–24	1.11	1.04–1.17	0.99	0.93–1.05	0.91	0.85–0.97
25–29	Ref.		Ref.		Ref.	
30–34	1.00	0.95–1.05	1.06	1.01–1.11	1.10	1.04–1.16
35–39	1.23	1.16–1.30	1.23	1.16–1.31	1.27	1.20–1.35
⩾40	1.61	1.46–1.78	1.44	1.30–1.60	1.47	1.32–1.63
First-trimester smoking						
No/missing	Ref.		Ref.		Ref.	
Sometimes	1.18	1.02–1.36	1.24	1.07–1.43	1.17	1.01–1.35
Daily	1.37	1.30–1.45	1.47	1.39–1.55	1.31	1.24–1.39
Chronic hypertension	4.30	3.76–4.89	2.10	1.83–2.41	2.07	1.80–2.37
Diabetes						
Type 1	4.60	4.01–5.27	2.71	2.35–3.13	2.66	2.30–3.07
Type 2	2.60	2.00–3.38	1.51	1.15–1.98	1.44	1.09–1.89
Gestational diabetes	1.47	1.30–1.68	1.07	0.94–1.22	1.05	0.92–1.20
PE/eclampsia/HELLP	10.21	9.76–10.68	9.63	9.19–10.09	9.52	9.08–9.98
Gestational hypertension	1.24	1.09–1.41	1.59	1.39–1.81	1.59	1.39–1.81
Placenta previa	11.95	10.62–13.45	13.56	12.00–15.33	13.45	11.90–15.21
IVF treatment	1.90	1.72–2.10	1.53	1.38–1.70	1.53	1.38–1.70
Parity						
Nulliparous	1.62	1.55–1.70	1.44	1.37–1.50	1.47	1.40–1.54
Para 1	Ref.		Ref.		Ref.	
Para >1	1.30	1.22–1.37	1.14	1.08–1.21	1.10	1.04–1.17
Previous stillbirth	3.05	2.69–3.45	2.91	2.55–3.32	2.90	2.54–3.31
Previous second-trimester abortion	1.83	1.67–2.00	1.81	1.65–1.99	1.79	1.63–1.97

Model 1: Adjusted for all the variables except education and marital status.

Model 2: Adjusted for all the variables included education and marital status.

Women born in sub-Saharan Africa had a 1.29-fold increased OR for very preterm birth compared to Norwegian-born women, with education and marital status in model 2 explaining 55.3% of the increased OR in model 1. Women born in East Asia Pacific/Oceania had a 1.16-fold increased OR for very preterm birth compared to Norwegian-born women, with education and marital status explaining 33.3% of the increased OR in model 1.

### Late preterm birth

The late preterm birth rate (between 34^+0^ and 36^+6^ gestational weeks) was 3.8% (*n*=34,206) in the study population. Late preterm birth rates were highest among women born in South Asia (4.9%) and East Asia Pacific/Oceania (4.9%; Table I). Compared to Norwegian-born women, we observed the greatest OR increases for late preterm birth among women born in East Asia Pacific/Oceania (OR=1.34; 95% CI 1.27–1.42), South Asia (OR=1.34; 95% CI 1.25–1.43) and women with an unknown country of birth (OR=1.22; 95% CI 1.07–1.40; Table IV). Adjusting for maternal and obstetric risk factors (model 1) increased the late preterm birth OR for women who were born in South Asia, women who were born in East Asia Pacific/Oceania, or women who had an unknown country of birth by 18–23%. Comparing models 1 and 2 revealed that education and marital status explained 17.5% of the increased ORs for late preterm birth among women who were born in East Asia Pacific/Oceania, 25% among women born in South Asia, and 37 % among with an unknown country of birth.

**Table IV.** Associations of factors with late preterm birth (*N*_group_=34,206) reported as crude and adjusted ORs with 95% CIs (*N*_total_=896,371).

Maternal characteristic	Late preterm birth 34^+0^ to 36^+6^ weeks					
	Crude analyses		Multivariable regression analyses, model 1		Multivariable regression analyses, model 2	
	OR	95% CI	aOR_1_	95% CI	aOR_2_	95% CI
Birthplace, by region						
Norway	Ref.		Ref.		Ref.	
Europe, EEA	0.88	0.84–0.92	0.92	0.87–0.96	0.91	0.87–0.96
Europe, non-EEA/Transcaucasia/Central Asia	0.90	0.84–0.97	0.95	0.88–1.02	0.91	0.84–0.98
North America	0.74	0.61–0.90	0.77	0.64–0.93	0.79	0.65–0.95
Latin America/Caribbean	1.13	1.01–1.26	1.14	1.02–1.28	1.08	0.97–1.21
Middle East/North Africa	1.01	0.94–1.09	1.09	1.01–1.17	1.01	0.93–1.09
Sub-Saharan Africa	0.91	0.85–0.98	0.94	0.87–1.01	0.82	0.77–0.89
South Asia	1.34	1.25–1.43	1.40	1.31–1.50	1.30	1.21–1.39
East Asia Pacific/Oceania	1.34	1.27–1.42	1.40	1.33–1.49	1.33	1.25–1.41
Unknown country of birth	1.22	1.07–1.40	1.27	1.10–1.46	1.17	1.00–1.36
Educational level						
None/primary education	1.13	1.10–1.17			1.09	1.06–1.13
Secondary education	Ref.				Ref.	
Bachelor	0.83	0.81–0.86			0.86	0.83–0.88
Master’s/PhD	0.76	0.73–0.79			0.80	0.76–0.83
Missing	0.98	0.92–1.03			1.01	0.94–1.08
Marital status						
Married/cohabiting/partner	Ref.				Ref.	
Single/widowed	1.34	1.30–1.39			1.18	1.14–1.23
Maternal age (years)						
<20	1.51	1.41–1.61	1.29	1.21–1.38	1.10	1.03–1.18
20–24	1.14	1.10–1.17	1.06	1.03–1.10	0.99	0.96–1.03
25–29	Ref.		Ref.		Ref.	
30–34	0.95	0.93–0.98	0.99	0.96–1.02	1.02	0.99–1.05
35–39	1.10	1.06–1.13	1.09	1.05–1.13	1.13	1.09–1.17
⩾40	1.39	1.31–1.48	1.28	1.20–1.37	1.31	1.22–1.39
First-trimester smoking						
No/missing	Ref.		Ref.		Ref.	
Sometimes	1.01	0.92–1.10	1.01	0.92–1.10	0.97	0.88–1.05
Daily	1.27	1.23–1.31	1.29	1.25–1.33	1.18	1.14–1.22
Chronic hypertension	2.65	2.40–2.92	1.72	1.55–1.91	1.70	1.54–1.89
Diabetes						
Type 1	6.05	5.58-6.55	4.87	4.48–5.30	4.82	4.43–5.24
Type 2	3.28	2.84–3.79	2.59	2.22–3.00	2.49	2.15–2.90
Gestational diabetes	2.02	1.90–2.16	1.73	1.62–1.85	1.70	1.59–1.82
PE/eclampsia/HELLP	5.32	5.14–5.50	4.95	4.78–5.12	4.90	4.74–5.08
Gestational hypertension	1.44	1.34–1.55	1.57	1.46–1.68	1.57	1.46–1.68
Placenta Previa	12.16	11.13–13.29	13.39	12.23–14.65	13.31	12.16–14.58
IVF treatment	1.50	1.41–1.60	1.29	1.21–1.38	1.29	1.20–1.38
Parity						
Nulliparous	1.44	1.40–1.48	1.34	1.30–1.37	1.36	1.33–1.40
Para 1	Ref.		Ref.		Ref.	
Para >1	1.18	1.15–1.22	1.11	1.08–1.15	1.08	1.04–1.11
Previous stillbirth	2.62	2.42–2.85	2.65	2.44–2.89	2.64	2.43–2.88
Previous second-trimester abortion	1.39	1.31–1.47	1.38	1.30–1.47	1.37	1.29–1.45

Model 1: Adjusted for all the variables except education and marital status.

Model 2: Adjusted for all the variables included education and marital status.

The association between maternal country of birth and preterm birth decreased as a function of increasing gestational length for women born in sub-Saharan Africa. The association changed from a dramatic 66% increased OR for extremely preterm birth to 18% reduced adjusted OR for late preterm birth compared to women born in Norway. The association between maternal country of birth and preterm birth increased with increasing gestational length for women born in East Asia Pacific/Oceania from a 13% reduced OR for extremely preterm birth to a 33% increased aOR for late preterm birth compared to women born in Norway. The association between maternal country of birth and preterm birth remained stable for all gestational groups for women born in South Asia, with increased ORs ranging from 30% to 56% compared to women born in Norway.

### Other risk factors and preterm birth

The most important risk factors for preterm birth were diabetes, chronic hypertension, preeclampsia/eclampsia/HELLP, placenta previa and previous intrauterine foetal death. In the multivariable analyses, maternal and obstetric risk factors did not appear to provide substantive explanatory power for associations between maternal country of birth and preterm birth, whereas education and marital status, as proxies of maternal socio-economic status, explained around 20–40% of the increased rate of preterm birth.

## Discussion

We found increased risk for extremely preterm birth among women born in sub-Saharan Africa, women born in South Asia and women with an unknown country of birth.

Prior studies on preterm birth among women born in sub-Saharan Africa defining preterm birth as birth before gestational week 37 have not been able to identify their increased risk for extremely preterm birth [10,11]. To the best of our knowledge, our study is the first to explore preterm birth risk according to maternal country of birth for several preterm birth groups separately. Our finding indicating that women with an unknown country of birth had a threefold increased OR for extremely preterm birth compared to Norwegian-born women is novel. In most studies, cases with unknown/missing information are excluded, obviating the emergence of such a finding. Because Norway has meticulous population birth registration, we can be confident that these women were not born in Norway. Data on maternal country of birth were collected from SSB. Information is therefore available, even if women were pregnant upon arrival. Hence, the unknown country of birth group most likely comprises women with temporary residence. Our findings indicate that this group is particularly vulnerable in respect to insufficient antenatal care and co-morbidities.

Our findings of increased very preterm birth risk among women born in South Asia, East Asia Pacific/Oceania and sub-Saharan Africa are consistent with the results of a study conducted in Sweden reporting increased relative risk for early preterm birth (<32 completed gestational weeks) among women from sub-Saharan Africa, South Asia and East Asia [12]. Two studies from Italy and one from France reported a similarly increased preterm birth (before week 32) risk for African-born women but not for Asian-born women [13–15].

Our findings of increased late preterm birth risk among women born in South Asia and East Asia Pacific/Oceania are consistent with several prior studies assessing preterm birth before gestational week 37 in Norway [10,16,17] and two conducted in Sweden that had a distinct late preterm birth period (32–36 gestational weeks) [12,18]. However, several other studies, two form Italy and one from France, did not reveal an increased risk for late preterm birth for current residents born in Asia [13,14,19].

Preterm birth rate disparities among women born in different regions of the world who gave birth in Norway could not be explained by known maternal and obstetric risk factors. Our observation of an association between socio-economic factors and preterm birth fits well with the results of prior studies assessing associations between socio-economic factors (represented by education levels and maternal occupation) and preterm birth risk conducted in countries with social and health-care systems that are similar to those in Norway [20–22].

Associations between maternal origin and preterm birth may differ in relation to risk factors related to immigrant status, country of origin and receiving country. Notably, content and timing of antenatal care has been associated with preterm birth [23]. Norwegian antenatal care is highly standardised in terms of number of visits as well as content. All residents, including immigrants, are offered at least eight visits, including the second-trimester ultrasound. Women with complicated pregnancies are offered additional care in hospital, all free of charge. Immigrant women who are pregnant upon arrival are immediately enrolled in the programme for antenatal care. Even though antenatal care is available for all pregnant women, the quality of care might be affected by language barriers and cultural views on health and pregnancy [24]. Our findings indicate that pregnant women in specific immigrant groups may benefit from customised antenatal care.

The mechanisms underlying the presently reported associations between maternal country of birth and preterm birth rates are unknown. Multiple factors may contribute. For example, preterm birth has been reported to be associated with some infections that have different incidence rates among women from different regions of the world [25], and some subpopulations are more prone to having nutritional deficits that have been associated with preterm birth [26]. Additionally, psychosocial stress has been associated with preterm birth, and immigrant women may have relatively elevated stress levels due to pre-migration factors or being pregnant in a new home country [27]. Moreover, genomic, epigenomic, microbiomic and metabolomic biomarkers may reveal contributors to differences in preterm birth requiring further investigation [28,29]. The main strength of the present study is its very large sample size, which enabled us to detect an association between the extremely preterm birth outcome and maternal country of birth. A secondary strength is that the study was population based. The MBRN is considered to be a reliable data source and suitable for research [30]. In addition, we can have high confidence in our gestational age data owing to the high coverage of comprehensive ultrasound examinations in Norway.

With respect to limitations, potential registry data errors are possible. However, underreporting is the most likely type of error. Such underreporting does not cause false associations, but it could weaken statistical associations between variables and outcomes. Second, maternal medical histories of previous preterm births and infections are not registered in the MBRN. Therefore, we could not assess these risk factors as confounders.

## Conclusions

Maternal country of birth was associated with preterm birth among women giving birth in Norway, even after adjusting for socio-economic factors and well-known other maternal and obstetric factors. Compared to Norwegian-born women, women with an unknown country of birth and women born in sub-Saharan Africa were at increased risk for extremely preterm birth, whereas women born in South Asia were at increased risk for preterm birth in all gestational age groups. These results suggest that some immigrant women may benefit from targeted surveillance and interventions during pregnancy, such as cervical length measurements or progesterone administration during pregnancy.
